# Oleuropein Protects Cardiomyocyte against Apoptosis via Activating the Reperfusion Injury Salvage Kinase Pathway In Vitro

**DOI:** 10.1155/2017/2109018

**Published:** 2017-04-13

**Authors:** Qiming Zhao, Yinliang Bai, Caie Li, Kun Yang, Wansheng Wei, Zhenzhen Li, Li Pan, Xiaoming Li, Xuanfen Zhang

**Affiliations:** ^1^Department of Cardiac ICU, Lanzhou University Second Hospital, Lanzhou 730030, China; ^2^Department of Pharmacy, Lanzhou University Second Hospital, Lanzhou 730030, China; ^3^Department of Pathology, Lanzhou University Second Hospital, Lanzhou 730030, China; ^4^Department of Orthopedic Surgery, Lanzhou University Second Hospital, Lanzhou 730030, China

## Abstract

Oleuropein, the main glycoside present in olives, has been reported to have cardioprotective effect, but the exact mechanism has not been clearly elucidated. This study attempted to clarify the cardioprotective effect of oleuropein against simulated ischemia/reperfusion- (SI/R-) induced cardiomyocyte injury in vitro and further explore the underlying mechanism. Here we confirmed that oleuropein reduced the cell injury in neonatal rat cardiomyocyte induced by SI/R evidenced by decreasing MTT dye reduction and LDH activity in the culture medium. Meanwhile, the compound also inhibited reactive oxygen species excessive generation and stabilized mitochondrial membrane potential after SI/R. The flow cytometry assessment results indicated the inhibition of cellular apoptosis with oleuropein treatment. Furthermore, western blot analysis showed that oleuropein attenuated the expression of Cyt-C, c-caspase-3, and c-caspase-9, increased the Bcl-2/Bax ratio, and enhanced the phosphorylation of ERK1/2 and Akt after SI/R. However, the phosphorylation enhancement was partially abolished in the presence of LY294002 (PI3K inhibitor) and U0126 (ERK inhibitor). All these findings indicate that oleuropein has the protective potential against SI/R-induced injury and its protective effect may be partly due to the attenuation of apoptosis via the activation of the PI3K/Akt and ERK1/2 signaling pathways.

## 1. Introduction

Coronary heart disease is one of the leading causes of death and disability worldwide. The effects of coronary heart disease are usually attributable to the detrimental effects of acute myocardial ischemia-reperfusion injury [1–3]. Oxidative stress and apoptosis have been identified to play important roles in the process of myocardial reperfusion injury [4, 5]. The oxidative stress outburst in the first minutes of myocardial reperfusion causes myocardial injury and cardiomyocyte death through several different mechanisms which provide therapeutic targets at the onset of myocardial reperfusion [4]. Apoptosis is initiated during ischemia and continued into reperfusion over several hours, providing a potential second therapeutic window for myocardial reperfusion [6]. Exploring agents that target both oxidative stress and apoptosis may be an effective therapy for ischemia diseases.

Oleuropein, one of the representative predominant phenolic oleosides in* Olea europaea*, has been shown to display several pharmaceutical properties, including antioxidant, anti-inflammatory, and anticancer effects [7]. In addition, previous studies have identified the cardioprotective effect of oleuropein mediated by its antioxidant property in different kinds of models [8–12]. Furthermore, Campolo et al. have reported that oleuropein could represent a possible treatment against secondary events of intestinal ischemia/reperfusion injury through anti-inflammatory and antiapoptotic pathways [13]. Considering the similarities in the pathologic mechanisms underlying ischemia/reperfusion injury between cardiomyocyte and intestinal, we question if an antiapoptotic mechanism is also involved in the cardioprotective effect of oleuropein. To the best of our knowledge, there has been no investigation focusing on the antiapoptotic effect of oleuropein in SI/R-induced cardiomyocytes injury.

Therefore, in the current study, we tested oleuropein with neonatal rat cardiomyocytes cells subjected to SI/R in vitro to investigate its cardioprotective effect and further explore the possible underlying mechanisms related to antiapoptotic property.

## 2. Materials and Methods

### 2.1. Chemicals and Reagents

Oleuropein was supplied by Shanghai Pure One Biotechnology (Shanghai, China) (purity > 98%). It was dissolved in distilled water and stored at 4°C. LDH ELISA kit and Annexin V-FITC Apoptosis Detection Kit were obtained from KeyGEN Biotech Ltd. (Nanjing, China). ROS Detection Kit was purchased from Beyotime Institute of Biotechnology (Nanjing, China). Dulbecco's Modified Eagle's Medium (DMEM) and other cell culture supplies were purchased from HyClone (Beijing, China). The rabbit anti-caspase-3, anti-caspase-9, anti-Bcl-2, anti-Bax, anti-Cyt-C, anti-p-Akt, anti-p-ERK, and *β*-actin antibodies were purchased from Santa Cruz Biotechnology (Santa Cruz, CA, USA). Complete Protease, PhosSTOP, and albumin from bovine serum (BSA) were obtained from Roche Molecular Biochemicals (Mannheim, Germany). BCA Protein Detection Kit and other materials for western blot were purchased from DingGuo Biotech Ltd. (Beijing, China). LY294002 (PI3K inhibitor) and U0126 (ERK inhibitor) were purchased from Sigma (Sigma, USA). Unless indicated otherwise, all chemicals were from Sigma.

### 2.2. Primary Culture of Cardiomyocytes

The experimental procedures were approved by the local Committee on Animal Care and Use. Every effort was made to minimize the number and suffering of animals in the following experiments. Neonatal rat cardiomyocytes were isolated from 1–3-day-old Sprague-Dawley rats. Briefly, rats were disinfected in 75% ethanol for 10 s and the hearts were removed. After washing with cold PBS, the ventricles were cut into small tissue blocks (1-2 mm^3^) and then digested with pancreatin at 37°C for 8 min with gentle shaking. Discarding the initial supernatant, the precipitates were digested with pancreatin until all tissues were completely digested. Finally, cells were filtered and suspended in DMEM medium containing 10% fetal bovine serum and maintained at 37°C in a 5% CO_2_ environment.

### 2.3. SI/R Injury Model In Vitro

To mimic the ischemic injury in vitro, ischemia and reperfusion were performed based on the method previously described [14]. The neonatal rat cardiomyocytes were incubated with ischemic buffer in a humidified atmosphere of 5% CO_2_ and 95% nitrogen for 4 h to simulate myocardial ischemia. The buffer consisted of 137 mM NaCl, 12 mM KCl, 0.49 mM MgCl_2_, 0.9 mM CaCl_2_•2H_2_O, 4 mM HEPES, and 20 mM sodium lactate (pH 6.2). After simulated ischemia, cells were reoxygenated in a standard incubator utilizing medium replacement with reoxygenation buffer for 24 h. The neonatal rat cardiomyocytes were randomly exposed to one of the following treatments during reperfusion: control, vehicle, oleuropein (100 *µ*g/mL, 200 *µ*g/mL, and 400 *µ*g/mL), oleuropein plus the PI3K inhibitor LY294002 [15], and oleuropein plus the ERK inhibitor U0126 [16]. The dosage of oleuropein was based on data previously published by Han et al. [17].

### 2.4. Measurement of Cellular Viability

Cellular viability was measured by MTT assay. Neonatal rat cardiomyocytes were seeded into a 96-well plate and treated with drugs under different conditions. After experimental treatment, MTT was added to each well (final concentration 0.5 mg/mL). Cells were incubated for 4 h at 37°C. The supernatants were aspirated, then the formazan crystals in each well were dissolved in 150 *μ*L DMSO, and the optical density at 570 nm was read on a microplate reader. The reduction in optical density was related to the percentage of viable cells indirectly.

### 2.5. Measurement of Cellular Injury In Vitro

Cellular injury was measured by LDH release. Culture medium of cardiomyocytes was collected after SI/R injury and the LDH activity was assayed with a microplate reader. Standard techniques were performed via using LDH ELISA kit according to the manufacturer's instructions. Cellular LDH release was expressed as the percentage of total cell LDH activity.

### 2.6. Measurement of Apoptosis by Flow Cytometry

Cell apoptosis was measured by fluorescein isothiocyanate- (FITC-) conjugated Annexin V and propidium iodide (PI) assay performed according to the manufacturer's instructions. Briefly, cells were washed twice with phosphate buffered saline (PBS) and then suspended in binding buffer. After incubation with FITC-Annexin V and PI for 10 min in the dark at room temperature, cell apoptosis was detected by flow cytometry for 1 h and analyzed by Cell Quest Pro software.

### 2.7. Measurement of Intracellular ROS Levels

ROS generation was measured by fluorescent probe 2′,7′-dichlorofluorescein diacetate (DCFH-DA) assay. Cells were washed twice with PBS and then incubated with 10 *µ*mol/L DCFH-DA for 20 min at 37°C. Cells were then washed three times with serum-free DMEM to remove extracellular DCFH-DA. Fluorescence intensity was measured by flow cytometry at excitation wavelength 488 nm and emission wavelength 525 nm.

### 2.8. Measurement of MMP

MMP was measured by cationic dye JC-1. JC-1 accumulates in the mitochondria in a potential-dependent manner. In normal mitochondria, JC-1 polymers produce strong red fluorescence in the mitochondria matrix. In unhealthy mitochondria, JC-1 accrues in the cytosol in monomer form producing green fluorescence. Cells were washed twice with PBS and then incubated with 10 mg/mL JC-1 for 15–20 min at 37°C. The cells were washed three times before they were suspended in incubation buffer and then analyzed by flow cytometry. Mitochondrial depolarization degree can be expressed as the ratio of green/red fluorescence intensity.

### 2.9. Western Blot Analysis

The total proteins were extracted from cultured neonatal rat cardiomyocytes and western blots were performed according to the manufacturer's procedures. In brief, equal samples from each group were loaded onto and separated by SDS polyacrylamide gel electrophoresis and transferred onto PVDF membrane. The membranes were blocked in 5% BSA for 1 h at room temperature and then incubated with primary antibody (c-caspase-3, Cyt-C, c-caspase-9, Bcl-2, Bax, p-ERK1/2, t-ERK1/2, p-Akt, t-Akt, and *β*-actin) overnight at 4°C, followed by secondary antibody conjugated to horseradish peroxidase for 2 h. Immunoblot was visualized with ECL western-blotting detection reagents and analyzed with Image pro plus V7.0 software.

### 2.10. Statistical Analysis

All data were analyzed by One-Way Analysis of Variance (ANOVA) using SPSS 16.0 software. Multiple comparison post hoc tests between groups were performed with Least-Significant Difference test. Data were presented as mean ± SE, and differences between groups were considered significant at *P* < 0.05.

## 3. Results

### 3.1. Oleuropein Ameliorated Cell Death In Vitro after SI/R

**Figure 1:**
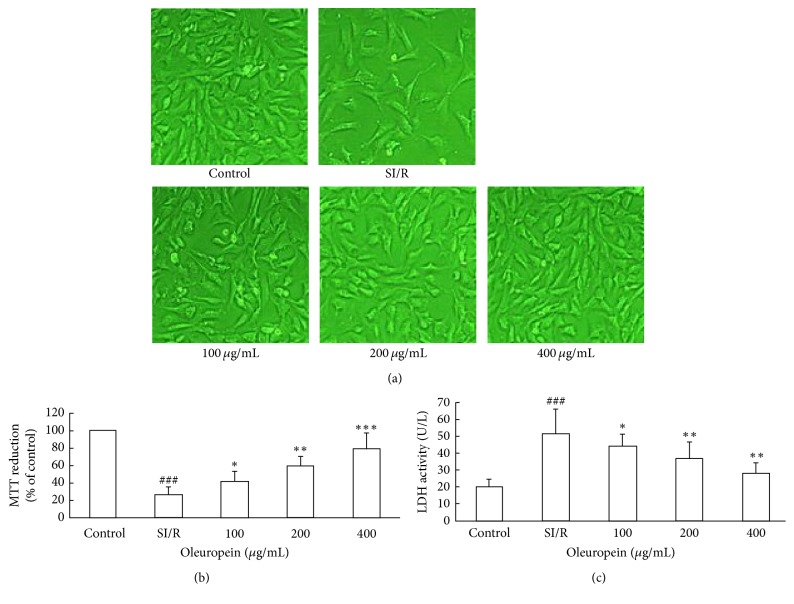
Oleuropein ameliorated neonatal rat cardiomyocytes death in vitro after SI/R. (a) Representative micrographs for cell after SI/R taken by an inverted light microscope (magnification 200x). (b) Cellular viability after SI/R was determined by MTT dye reduction. (c) The LDH activity in culture medium at the end of reperfusion was determined. All data were presented as mean ± SE (*n* = 4). SI/R: simulated ischemia/reperfusion.^  ###^*P* < 0.001 versus control; ^*∗*^*P* < 0.05, ^*∗∗*^*P* < 0.01, and ^*∗∗∗*^*P* < 0.001 versus SI/R.

Firstly, we confirmed the cardioprotective effect of oleuropein in neonatal rat cardiomyocytes in vitro using MTT assay. We found MTT dye reduced obviously after neonatal rat cardiomyocytes were subjected to SI/R injury. However, treatment with oleuropein (100, 200, and 400 *µ*g/mL) significantly increased the MTT dye reduction from 25.6 ± 9.6% to 41.2 ± 12.4%, 59.1 ± 10.3%, and 78.6 ± 18.1% in a dose dependent manner, as shown in Figures 1(a) and 1(b). The results convinced the ability of oleuropein to protect the neonatal rat cardiomyocytes against SI/R injury.

LDH leakage is also an index of neonatal rat cardiomyocytes injury. LDH is retained in the cytosol under normal circumstances. Once the sarcolemma membrane is ruptured, LDH diffuses into the surrounding media. We measured LDH activity in the culture medium at the end of reperfusion. Compared to control group, the level of LDH was significantly elevated in the SI/R treatment group. Treatment with oleuropein reduced the LDH activity in a dose dependent manner (Figure 1(c)).

### 3.2. Oleuropein Reduced Intracellular ROS Generation in Neonatal Rat Cardiomyocytes Subjected to SI/R

**Figure 2:**
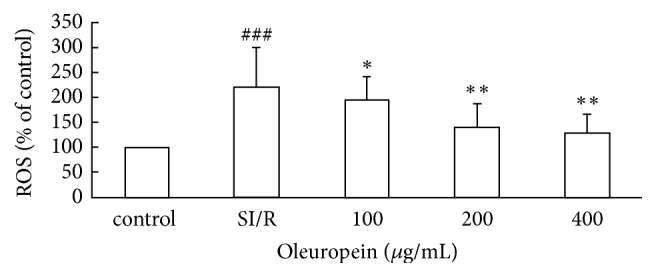
Oleuropein reduced intracellular ROS generation in neonatal rat cardiomyocytes subjected to SI/R. Intracellular ROS levels were assessed by determining DCF fluorescence intensity via flow cytometry. All data were presented as mean ± SE (*n* = 4). SI/R: simulated ischemia/reperfusion. ^  ###^*P* < 0.001 versus control; ^*∗*^*P* < 0.05, ^*∗∗*^*P* < 0.01 versus SI/R.

Intracellular ROS level was assessed by determining DCF fluorescence intensity via flow cytometry (Figure 2). After 24 h reperfusion, we detected strong DCF fluorescence intensity in the SI/R treatment group, indicating the intracellular ROS outburst after SI/R injury. The value of DCF fluorescence intensity was increased to 2.2-fold change when compared with the control cells. However, all dosage of oleuropein significantly inhibited the increase of ROS, suggesting its antioxidant effect.

### 3.3. Oleuropein Inhibited Apoptosis Induced by SI/R in Neonatal Rat Cardiomyocytes

**Figure 3:**
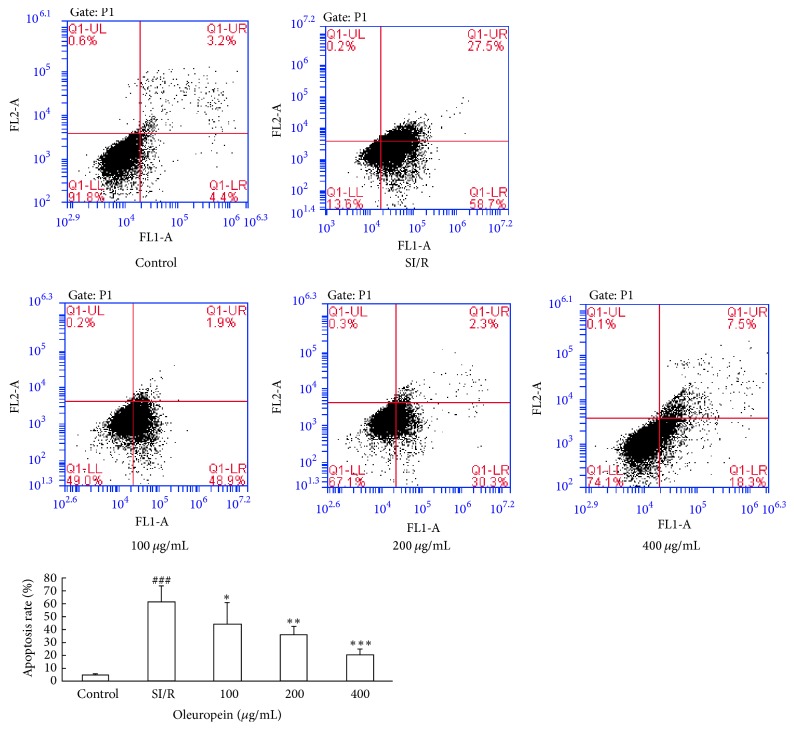
Oleuropein inhibited apoptosis induced by SI/R in neonatal rat cardiomyocytes. SI/R-induced apoptosis was determined by Annexin V-FITC/PI flow cytometry in control and SI/R groups with or without oleuropein. All data were presented as mean ± SE (*n* = 4). SI/R: simulated ischemia/reperfusion. ^  ###^*P* < 0.001 versus control; ^*∗*^*P* < 0.05, ^*∗∗*^*P* < 0.01, and ^*∗∗∗*^*P* < 0.001 versus SI/R.

To assess the apoptotic inhibitory effect of oleuropein, cellular apoptosis was assessed by flow cytometry. As shown in Figure 3, the apoptosis rate of control cells was only 4.7 ± 0.6%. SI/R induced a significant increase in apoptosis rate to 61.3 ± 12.3% when compared to control cells, while oleuropein treatment markedly decreased apoptosis rate by 44.5 ± 16.4%, 35.7 ± 6.8%, and 20.1 ± 4.9% at 100 *µ*g/mL, 200 *µ*g/mL, and 400 *µ*g/mL dose, respectively, showing its antiapoptotic property in neonatal rat cardiomyocytes.

### 3.4. Oleuropein Stabilized MMP in Neonatal Rat Cardiomyocytes Subjected to SI/R

**Figure 4:**
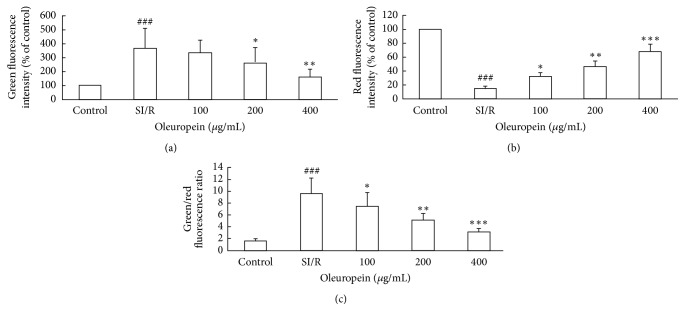
Oleuropein steadied MMP in neonatal rat cardiomyocytes subjected to SI/R. MMP was measured with fluorescent dye JC-1 via flow cytometry. Magnitude of mitochondrial depolarization was related to the ratio of red/green fluorescence intensity. (a) Green fluorescence intensity after SI/R. (b) Red fluorescence intensity after SI/R. (c) Green/red fluorescence intensity ratio after SI/R. All data were presented as mean ± SE (*n* = 4). SI/R: simulated ischemia/reperfusion. ^  ###^*P* < 0.001 versus control; ^*∗*^*P* < 0.05, ^*∗∗*^*P* < 0.01, and ^*∗∗∗*^*P* < 0.001 versus SI/R.

MMP is an important early marker of the mitochondrial apoptotic pathway. We investigated the effect of oleuropein upon MMP by cationic dye JC-1. In comparison with control cells, the green fluorescence intensity of SI/R treated cells was obviously enhanced and red fluorescence intensity diminished instead; hence the ratio of green/red fluorescence intensity was elevated significantly. Oleuropein treatment reversed all of these effects, as described in Figure 4.

### 3.5. Oleuropein Modulated Apoptotic Proteins in Neonatal Rat Cardiomyocytes Subjected to SI/R

**Figure 5:**
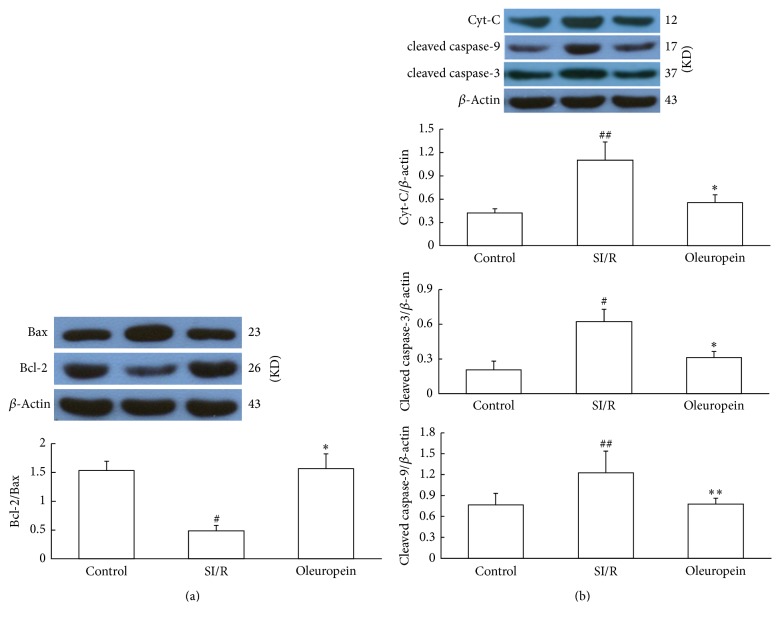
Oleuropein modulated Bcl-2, Bax, cleaved caspase-3, cleaved caspase-9, and Cyt-C expression in neonatal rat cardiomyocytes subjected to SI/R. (a) SI/R upregulated Bax expression and downregulated Bcl-2 expression, hence decreasing the Bcl-2/Bax ratio compared to the control cells, but 400 *µ*g/mL oleuropein increased the Bcl-2/Bax ratio. (b) SI/R significantly increased expression of Cyt-C, cleaved caspase-3, and cleaved caspase-9, which were attenuated by 400 *µ*g/mL oleuropein treatment. All data were presented as mean ± SE (*n* = 4). SI/R: simulated ischemia/reperfusion. ^#^*P* < 0.05, ^##^*P* < 0.01 versus control; ^*∗*^*P* < 0.05, ^*∗∗*^*P* < 0.01 versus SI/R.

To further study the molecular mechanism underlying oleuropein-mediated cardioprotection, we assayed apoptotic related proteins in neonatal rat cardiomyocytes after SI/R by western blot analysis.  Figure 5(a) illustrated that SI/R injury induced upregulation of Bax expression and downregulation of Bcl-2 expression, thereby decreasing the Bcl-2/Bax ratio. However, 400 *µ*g/mL oleuropein treatment elevated the Bcl-2/Bax ratio when compared to the SI/R group. In addition, SI/R injury significantly increased the expression of Cyt-C, c-caspase-3, and c-caspase-9, but these increments were attenuated by 400 *µ*g/mL oleuropein treatment (Figure 5(b)). The western blot results have given molecular evidences for the antiapoptotic property of oleuropein in vitro.

### 3.6. Oleuropein Increased Phosphorylation of Akt and ERK1/2 in Neonatal Rat Cardiomyocytes Subjected to SI/R

**Figure 6:**
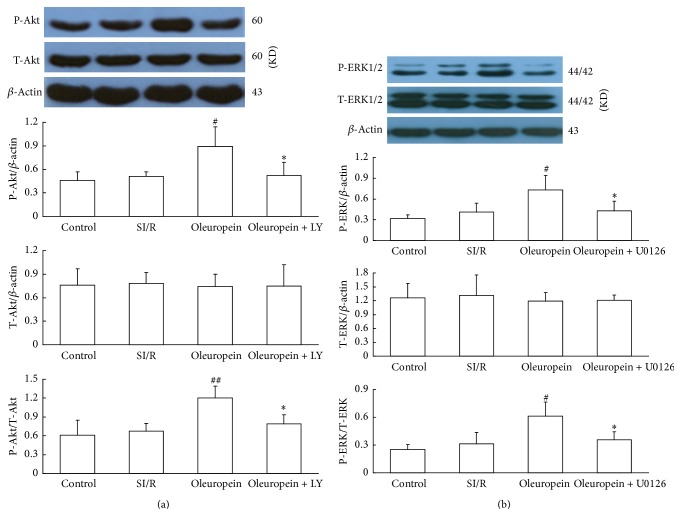
Oleuropein increased phosphorylation of Akt (a) and ERK1/2 (b) in neonatal rat cardiomyocytes subjected to SI/R. The effects of oleuropein were blocked by LY294002 (PI3K inhibitor) or U0126 (ERK inhibitor). Total ERK1/2 and Akt levels were not significantly different among the groups. All data were presented as mean ± SE (*n* = 4). SI/R: simulated ischemia/reperfusion. ^#^*P* < 0.05, ^##^*P* < 0.01 versus control; ^*∗*^*P* < 0.05 versus SI/R.

We also analyzed whether the antiapoptotic effect of oleuropein was mediated by Reperfusion Injury Salvage Kinase (RISK) pathway. As shown in Figure 6, the total ERK1/2 and Akt levels had no significant differences among all groups, yet 400 *µ*g/mL oleuropein enhanced the phosphorylation of ERK1/2 and Akt after SI/R. However, when they were simultaneously incubated with LY294002 (PI3K inhibitor) or U0126 (ERK inhibitor), the enhancement of ERK1/2 and Akt phosphorylation effects induced by oleuropein was blocked.

## 4. Discussion

Although improvements in myocardial reperfusion continue to take place in terms of new antiplatelet and antithrombotic agents, there is still no effective therapeutic strategy for preventing myocardial reperfusion injury. Many people have focused attention on natural products which may be a viable approach to develop safe and effective treatments [18].

In the present study, we demonstrated that oleuropein, the main glycoside present in olives, attenuated cell injury in the neonatal rat cardiomyocytes subjected to SI/R as evidenced by decreasing MTT dye reduction and LDH activity in the culture medium. Since MTT dye is reduced by decreased active mitochondria in living cells, we cannot confirm that the reduction resulted from the decrement of cells or the decline of dehydrogenase activity. Here, we also tested the whole activity of LDH in cells after SI/R to reflect the cell viability. However, LDH muscle subunit (M-LDH) released from cardiomyocytes after SI/R has a role in protecting the heart from oxidative stress-induced injury through an intracellular signal transduction pathway involving ERK1/2 [19]. Further study should be carried out to explore the changing in each subunit of LDH after SI/R.

Then we further discovered that treatment with oleuropein could reduce generation of ROS in cardiomyocytes, stabilize the MMP, and inhibit myocardial apoptosis induced by SI/R. As we all know, oxidative stress and apoptosis have been reported to play major roles in the progression of myocardial infarction, ischemia/reperfusion, hypertension, cardiomyopathies, and atherosclerosis [20, 21]. Ischemia and reperfusion disturb the balance of ROS and the endogenous antioxidant system, which increases oxidative stress by the overproduction of cellular ROS [22, 23]. Previous studies examined the mechanism of cardioprotective effect of oleuropein and suggested that the beneficial effect of oleuropein may be partly attributed to the inhibition of oxidative stress [8–10]. Here, we also found that treatment with oleuropein could reduce generation of ROS in cardiomyocytes after SI/R injury, which was in accordance with the above literatures.

Cumulative evidences suggest that ROS, implicated in reperfusion toxicity, can trigger cardiomyocyte apoptosis via the mitochondrial apoptosis pathway [24, 25]. During intracellular ROS overproduction, collapse of the MMP results in the loss of integrity of the outer mitochondrial membrane, releasing mitochondrial protein from mitochondrial matrix to cytoplasm. The release of cytochrome c along with complete loss of membrane potential causes apoptosis enzyme cascade effects [26]. Caspases transduce and execute apoptotic signaling [27]. On the other hand, as a double-edged sword, apoptosis in the remote noninfarcted myocardium may be partly responsible for myocardial remodelling and dilatation after myocardial infarction and may be amenable to treatment, which is frequently followed by secondary necrosis of cells, especially if there is failure of clearance or ingestion of apoptotic bodies [28, 29]. In the present study, we found oleuropein treatment mitigated SI/R-induced apoptosis and stabilized the MMP in neonatal rat cardiomyocytes. Western blot results further supported our findings: oleuropein significantly increased the Bcl-2/Bax ratio and decreased the expression of Cyt-C, c-caspase-3, and c-caspase-9.

Hausenloy and Yellon use the term RISK pathway to represent the PI3K-Akt and ERK1/2 prosurvival kinase cascades that have been implicated in protecting the heart against cell death during the reperfusion phase [30]. Previous studies have illustrated cardioprotective effects of many drugs through the activation of RISK pathway [31–34]. As one of the key roles in RISK pathway, Akt inhibits apoptosis by reducing the release of cytochrome c from mitochondria and stabilizing the MMP induced by multiple apoptotic stimuli [35]. Furthermore, the change of Bcl-2/Bax ratio may be correlated with the activation of PI3K/Akt [36]. On the other hand, ERK1/2 kinase activation is able to inhibit cytochrome c-induced caspase activation possibly by inactivating one component of the caspase cascade [37, 38]. Through the activation or inhibition of its downstream target proteins, the RISK pathway is directly related to the activation of the protective mechanism that reduces the apoptosis of cardiomyocytes. In the present study, our results showed that oleuropein enhanced the phosphorylation of ERK1/2 and Akt and attenuated cellular apoptosis compared with that of SI/R group. Furthermore, LY294002 (PI3K inhibitor) or U0126 (ERK inhibitor) partially blocked the effects of oleuropein, suggesting that the antiapoptotic effect of oleuropein was PI3K/Akt and ERK1/2 dependent.

Taken together, we confirmed the cardioprotective effect of oleuropein in neonatal rat cardiomyocytes subjected to SI/R in vitro and demonstrated that oleuropein protected against myocardial SI/R injury by both reducing intracellular ROS and inhibiting apoptosis. For the first time, our results suggested that activation of RISK pathway was involved in the cardioprotective effect of oleuropein. However, further studies in vivo are required to identify the specific molecular targets of oleuropein.
